# Intestinal B^0^AT1 (SLC6A19) and PEPT1 (SLC15A1) mRNA levels in European sea bass (*Dicentrarchus labrax*) reared in fresh water and fed fish and plant protein sources

**DOI:** 10.1017/jns.2015.9

**Published:** 2015-05-20

**Authors:** Simona Rimoldi, Elena Bossi, Sheenan Harpaz, Anna Giulia Cattaneo, Giovanni Bernardini, Marco Saroglia, Genciana Terova

**Affiliations:** 1 Department of Biotechnology and Life Sciences, University of Insubria, 21100 Varese, Italy; 2 Agricultural Research Organization, The Volcani Center, Bet Dagan 50250, Israel; 3 Inter-University Centre for Research in Protein Biotechnologies, “The Protein Factory”, Polytechnic University of Milan and University of Insubria, Varese, Italy

**Keywords:** Aquaculture, Gene expression, Real-time PCR, Fish meal replacement, Vegetable meal, Dietary salt addition, ACE2, angiotensin-converting enzyme 2, FCR, feed conversion ratio, FM, fish meal, PEPT1, oligopeptide transporter 1, RACE, rapid amplification of the cDNA ends, SBM, soyabean meal, SGR, specific growth rate, SLC6A19, solute carrier family 6 member 19

## Abstract

The objective of the present study was to examine the effect of diets with descending fish meal (FM) inclusion levels and the addition of salt to the diet containing the lowest FM level on growth performances, feed conversion ratio, and intestinal solute carrier family 6 member 19 (*SLC6A19*) and oligopeptide transporter 1 (*PEPT1*) transcript levels, in freshwater-adapted European sea bass (*Dicentrarchus labrax*). We first isolated by molecular cloning and sequenced a full-length cDNA representing the neutral amino acid transporter SLC6A19 in sea bass. The cDNA sequence was deposited in GenBank database (accession no. KC812315). The twelve transmembrane domains and the ‘*de novo*’ prediction of the three-dimensional structure of SLC6A19 protein (634 amino acids) are presented. We then analysed diet-induced changes in the mRNA copies of *SLC6A19* and *PEPT1* genes in different portions of sea bass intestine using real-time RT-PCR. Sea bass were fed for 6 weeks on different diets, with ascending levels of fat or descending levels of FM, which was replaced with vegetable meal. The salt-enriched diet was prepared by adding 3 % NaCl to the diet containing 10 % FM. *SLC6A19* mRNA in the anterior and posterior intestine of sea bass were not modulated by dietary protein sources and salt supplementation. Conversely, including salt in a diet containing a low FM percentage up-regulated the mRNA copies of *PEPT1* in the hindgut. Fish growth correlated positively with the content of FM in the diets. Interestingly, the addition of salt to the diet containing 10 % FM improved feed intake, as well as specific growth rate and feed conversion ratio.

Fish meal (FM) and fish oil are the preferred ingredients in fish feeds because they: (a) contain all the nutrients required for farmed fish to grow in nearly perfect balance; (b) are easily digestible by the fish; (c) result in good growth and survival; and (d) provide human health benefits. However, FM supply has become a limiting factor for the further development of fish feed production. Increased FM costs, together with the limited availability, has encouraged the use of alternative sustainable sources of protein in fish feeds in the last few decades^(^^1^^,^^2^^)^. Thus, high rates of several terrestrial plant meals have been successfully included in the feed without affecting fish growth and production quality^(^^3^^–^^5^^)^. Nevertheless, plant protein sources differ greatly in their nutritional value, not only regarding their amino acid profiles and digestibility, but also their carbohydrate content and characteristics and processing methods^(^^6^^–^^9^^)^. Therefore, the degree of success differs for different types of plant proteins. Of all plant products used in fish farming, soyabeans are the most promising because of their higher protein content, higher digestibility and better amino acid profile than other grains and oilseeds^(^^7^^)^. Therefore, solvent-extracted soyabean meal (SBM) is currently considered to be one of the most suitable and stable alternative ingredients for replacing FM in commercial fish feeds.

To be efficiently absorbed by the fish intestine, dietary proteins must be hydrolysed to yield di- and tri-peptides and free amino acids. Inside the enterocyte, peptides are then hydrolysed, and the resulting amino acids are released into the bloodstream together with those absorbed by amino acid transporters^(^^10^^)^.

Oligopeptides and free amino acids are absorbed along the intestinal tract by specialised membrane transporter proteins. Di- and tri-peptides are transported via H^+^/coupled peptide transporter 1 (PEPT1), which is a member of the proton oligopeptide cotransporter family^(^^11^^)^ located in the brush-border membrane of intestinal epithelial cells. Free amino acids are absorbed by a variety of Na^+^-dependent and -independent membrane transporters^(^^12^^)^, frequently referred to as ‘systems’. Five amino acid transport activities have been proposed^(^^10^^)^: (1) the ‘neutral system’ or ‘methionine-preferring system’ transporting all neutral amino acids; (2) the ‘basic system’ transporting cationic amino acids together with cystine; (3) the ‘acidic system’ transporting glutamate and aspartate; (4) the ‘iminoglycine system’ transporting proline, hydroxyproline and glycine; and (5) the β-amino acid system. Among the amino acid-transporting proteins, the neutral amino acid transporter solute carrier family 6 member 19 (SLC6A19), also called system B^0^ neutral amino acid transporter AT1 (B^0^AT1), is an integral plasma membrane protein responsible for the uptake of a broad range of neutral amino acids across the apical membrane of enterocytes and renal cells. Initially cloned from mouse kidney^(^^13^^)^, the product of the *SLC6A19* gene was identified as a neutral amino acid transporter belonging to the family of Na^+^ and Cl^–^-dependent neurotransmitter transporters (SLC6)^(^^14^^,^^15^^)^. SLC6A19 actively transports most large neutral amino acids, but not anionic or cationic ones, against a concentration gradient, from the lumen into the epithelial cells of the small intestine and kidney. Transporting substrates across the membrane is coupled with the Na^+^ electrochemical gradient^(^^13^^,^^15^^)^. SLC6A19 carries all neutral amino acids in the following order of preference: Leu = Val = Ile = Met > Gln = Phe = Ala = Ser = Cys = Thr > His = Trp = Tyr = Pro = Gly^(^^10^^,^^13^^)^. In mammals, SLC6A19 expression appears to be largely restricted to the brush-border membrane of small-intestine enterocytes and to the early portion of proximal kidney tubules, which are the major sites for the absorption and reabsorption of nutrients in the body, respectively^(^^10^^,^^16^^)^. Although the kinetic properties and substrate specificity of SLC6A19 have been extensively studied^(^^14^^,^^17^^)^, at present, very little is known about the mechanisms regulating its activity and expression. SLC6A19 interacts with two tissue-specific accessory proteins: collectrin in the kidney and angiotensin-converting enzyme 2 (ACE2) in the small intestine. A strong down-regulation of SLC6A19 expression in the kidney and small intestine was observed in the collectrin and ACE2 knock-out animals, respectively. Additionally, a significant increase in transporter function was observed when SLC6A19 was coexpressed with collectrin or ACE2 in oocytes of *Xenopus laevis*^(^^18^^,^^19^^)^. Recently, Fairweather *et al.*^(^^20^^)^ discovered that, in addition to its known interaction with ACE2, mouse SLC6A19 also forms functional complexes with the peptidase APN (aminopeptidase N/CD13). These complexes are likely to increase the efficiency of protein absorption by increasing the local substrate concentration of B^0^AT1.

In humans, the B^0^ system is associated with a severe neutral aminoaciduria known as Hartnup disorder, an autosomal recessive defect named after the English family in which it was described for the first time^(^^21^^)^. This disease causes impaired transport of neutral amino acids across epithelial cells in renal proximal tubules and intestinal mucosa; symptoms include transient manifestations of pellagra, cerebellar ataxia and psychosis^(^^13^^,^^22^^)^.

Nozaki *et al.*^(^^23^^)^, who performed homozygosis mapping and linkage analysis on consanguineous Japanese pedigrees and six Australian pedigrees, respectively, demonstrated linkage of Hartnup disorder to band 5p15. This region was found to be homologous to the area of mouse chromosome 13 that encodes the Na^+^-dependent amino acid transporter SLC6A19. Positional cloning and candidate gene analysis then definitely confirmed the chromosome region 5p15.33 and specifically the *SLC6A19* gene as the site of mutations causing Hartnup disorder^(^^24^^)^. However, in individuals affected by Hartnup disease, the reduced amino acid absorption through intestinal epithelium is partly compensated by the activity of another intestinal transporter, PEPT1^(^^25^^)^. PEPT1 is a low-affinity, high-capacity transporter that mediates electrogenic uphill transport of di- and tripeptides from the intestinal lumen into the enterocytes. The transport is energised by a transmembrane electrochemical H^+^ gradient directed from outside to inside. The nutritional importance of PEPT1 is related to its dual role: absorption of a remarkable range of dietary protein-derived substrates in the intestine and reabsorption of peptide-bound amino-N from glomerular filtrate in the kidney^(^^26^^)^.

The *PEPT1* gene has been characterised in many vertebrates, including fish, such as zebrafish (*Danio rerio*)^(^^27^^)^, sea bass (*Dicentrarchus labrax*)^(^^28^^)^, common carp (*Cyprinus carpio*)^(^^29^^)^, Atlantic salmon (*Salmo salar*)^(^^30^^)^, rainbow trout (*Onchorhynchus mykiss*)^(^^31^^)^, yellow perch (*Perca flavescens*)^(^^32^^)^ and sea bream (*Sparus aurata*)^(^^33^^)^. According to these studies, the *PEPT1* gene is specifically expressed in the intestinal tract and its transcript levels are related to fish nutritional status, which is down-regulated during fasting and up-regulated during refeeding^(^^28^^)^. However, the expression and regulation of *PEPT1* when vegetable sources are used as a substitute for FM has only been explored in very few fish species^(^^33^^)^ and the corresponding information for *SLC6A19* is completely unknown. Moreover, cDNA sequences of *SLC6A19* have been cloned in only three fish species so far: Atlantic salmon (*Salmo salar*) (GenBank accession number: NM_001141815), Nile tilapia (*Oreochromis niloticus*) (XM_003448888) and zebrafish (*Danio rerio*) (BC059804).

Accordingly, the aim of the present study was to provide knowledge on the transcriptional regulation of intestinal SLC6A19 and PEPT1 transporters in freshwater-adapted sea bass that were fed protein from plant sources. We firstly cloned and sequenced a full-length cDNA representing sea bass *SLC6A19*. The predicted transmembrane domains and the three-dimensional structure of the protein are presented. Then, we investigated the effect of six different diets with different percentages of FM replaced with SBM protein on *SLC6A19* and *PEPT1* mRNA levels in the proximal and distal intestine, with the aim to correlate gene expression profiles with fish feeding status.

*Dicentrarchus labrax* is a euryhaline fish that can survive in a wide range of salinities from fresh water to 150 % seawater salinity. The use of freshwater-adapted sea bass represents a novelty since most nutrition research on cultured marine fish species has been carried out in saltwater environments. The reason for this choice was to examine the beneficial effects of dietary salt supplementation in a low-FM diet. In freshwater-adapted fish, the passive outward flux of ions such as Na^+^ and Cl^–^ from the fish to the external medium must be overcome by active uptake of ions (for example, Na^+^, Cl^–^, K^+^ and Ca^2+^) from the water and/or from the diet^(^^34^^)^. Therefore, the diet constitutes an important source of salt that can satisfy the osmoregulatory requirements of fish kept in freshwater, thus making it possible to spare the energy used for osmoregulation^(^^35^^,^^36^^)^. Therefore, in feeds formulated for freshwater-adapted fish, the dietary mineral content represents a crucial factor that needs to be kept in mind, in particular when FM is replaced with various plant ingredients, which are notoriously poor in salt. Indeed, in feeds, a large percentage of salt originates from the FM furnished from the diet^(^^37^^)^.

## Materials and methods

### Feeds, fish and feeding protocol

The experiment was carried out at the Agricultural Research Organization, The Volcani Centre (Bet Dagan, Israel). The experimental procedures used in the present study were approved by the Animal Policy and Welfare Committee of the Agricultural Research Organization (approval number IL-241/10), Volcani Research Centre, and were in accordance with the guidelines of the Israel Council on Animal Care.

Juvenile European sea bass (*Dicentrarchus labrax*) were bought from a commercial hatchery (Maagan Michael, Israel). They were transported in brackish water (10 parts per trillion) and stocked in cages placed in a large 6000-litre concrete pond. After 1 week of acclimatisation, they were transferred to the experimental tanks and slowly acclimatised to fresh water (< 3 parts per trillion) over a period of 1 week.

At the beginning of the experiment and after removing those fish substantially deviating from the average weight (approximately 10 g), fifty fish were counted, bulk weighed, and stocked in each tank. There were no significant differences among the experimental tanks at the beginning of the trial (*P* > 0·05).

All rearing tanks were located in an indoor facility. The tanks were connected to a recirculating system and equipped with a computerised control system to regulate photoperiod and temperature. The light source was natural daylight enhanced with florescent light, providing a light intensity of 1200 lux during the daytime. The water was maintained at 24·0 ± 2·0 °C using submersible aquarium heaters.

The experimental layout consisted of three systems, each composed of six tanks of 250 litres and a central main biofilter of 350 litres. Six experimental diets were tested in triplicate (three tanks per each diet) so that each diet was represented in each system set. The feed consumption, fish mortality and tank temperature were registered daily. Twice per week dissolved oxygen, pH, ammonia and nitrite levels were measured. The fish were fed with six isonitrogenous diets, with ascending fat levels or descending FM levels, formulated and prepared in the form of sinking extruded pellets, 3 mm in diameter, by Raanan Feeds Ltd. The experimental salt-enriched diet was prepared by adding 3 % NaCl to the diet containing 10 % FM. The diets contained 45 % protein, 16–20 % fat, 1·7–2·3 % fibre and 6·1–8·2 % ash. In the diets with lower amounts of FM, the FM ingredient was replaced with defatted SBM and soya protein concentrate. The information concerning the different diets used is reported in Table 1.

**Table 1.** Proximate composition of the experimental diets, raw material and nutritional content of experimental diets

	Diet 1 (%)	Diet 2 (%)	Diet 3 (%)	Diet 4 (%)	Diet 5 (%)	Diet 6 (%)
Raw material						
Salt (pure NaCl)						3·0
Full-fat soya					12·8	13·5
Soya protein concentrate					13·6	16·5
Wheat	17·1	10·7	12·0	12·0	8·0	7·0
Dried distiller's grain with soluble	8·0	10·0	10·0	6·35		
Wheat gluten meal	4·0	4·0	4·0	5·3	8·19	10·0
Dicalcium phosphate					1·72	1·64
Mixed oil*	10·0	12·0	14·0	12·0	12	12·0
Lysine (98 %)					0·29	0·36
Vitamin and mineral premix†	0·4	0·4	0·4	0·4	0·4	0·4
Maize gluten		8·2	3·0	16·0	16·0	16·0
Soyabean meal (48 %)	10·6	5·0	6·6	18·0	16·7	9·44
Fish meal (65 %)	50·0	50·0	50·0	30·0	10·0	10·0
Anti-mould agent	0·1	0·1	0·1	0·1	0·1	0·1
Nutrients (%)						
Protein	45·0	45·0	45·0	45·0	45·0	45·0
Fat	16·0	18·0	20·0	16·0	16·0	16·0
Fibre	1·8	1·7	1·7	1·8	2·3	2·1
Ash	8·2	7·9	8·0	6·1	6·4	6·1
Ca	1·7	1·7	1·7	1·2	1·0	0·98
Total P	1·3	1·2	1·3	0·9	0·95	0·91
Methionine	1·1	1·2	1·2	1·1	0·9	0·9
Methionine + cysteine	1·6	1·6	1·6	1·7	1·6	1·6
Lysine	3·0	2·9	2·9	2·3	2·3	2·3

* Mixed oil: 40 % maize, 60 % soya.

† Vitamin and mineral premix (quantities in 1 kg of mix): vitamin A, 1200 mg; vitamin D_3_, 20 mg; vitamin C, 25000 mg; vitamin E, 15000 mg; inositol, 15000 mg; niacin, 12000 mg; choline chloride, 6000 mg; calcium pantothenate, 3000 mg; vitamin B1, 2000 mg; vitamin B3, 2000 mg; vitamin B6, 1800 mg; biotin, 100 mg; Mn, 9000 mg; Zn, 8000 mg; Fe, 7000 mg; Cu, 1400 mg; Co, 160 mg; I, 120 mg; anticaking agents, antioxidant and carrier, making up to 1000 g.

The fish were fed daily *ad libitum* for 6 weeks in the following manner. Feed for each tank was weighed separately and placed in a container. The feed from each container was given in the morning by hand, in small quantities until the fish ceased to respond. In the afternoon, the process was repeated. At the end of the day, feed left in each container was weighed and the feed consumption of each tank was then registered.

During the period of adaption from brackish water to fresh water and before the feeding trial, all the fish were fed the diet containing 50 % FM and 16 % fat (diet no. 1; control diet).

At the end of the feeding trial, each dietary group was batch weighed after overnight feed deprivation. From each tank, five fish were then randomly selected, anaesthetised using clove oil, and then killed; their proximal and distal intestines were dissected out using sterile instruments, frozen, and kept at –80 °C until further analysis.

In five fish from the control group, we dissected the gut into sections for regional analysis of the spatial distribution of *SLC6A19* mRNA along the gut axis. To ensure nutritional exposure of the enterocytes (thereby maximising likelihood of *SLC6A19* mRNA regulation) our fish were sampled 12 h postprandial. We removed the whole intestine by cutting right after the pyloric caeca and right before the anus. The intestine was then divided into ten equally long parts. The last segment (segment 10) also comprised the hindgut. We emptied the intestine of any leftover feed and chyme by gently stroking the content out and rinsed thoroughly each segment in a PBS solution (145 mm-NaCl, 8 mm-Na_2_HPO_4_, 2 mm-NaH_2_PO_4_, pH 7·2). Segments were then frozen in liquid N_2_ and stored at −80 °C until analysed. A sample of approximately 150 mg tissue was taken from the centre part of each segment for RNA isolation, using dry ice to prevent thawing. Other organs/tissues such as liver, gills, white muscle, brain, heart and kidney were dissected out for the same analysis, too.

Fish specific growth rate (SGR) was calculated using the following formula: (ln W_f_ – ln W_i_)/t × 100, where W_f_ is the final weight (g), W_i_ is the initial weight (g), and t is growth time (d).

### Molecular cloning, sequencing and gene expression

These analyses were carried out at the Department of Biotechnology and Molecular Sciences of the University of Insubria (Varese, Italy).

### Isolation of total RNA, cDNA synthesis and sea bass *SLC6A19* cloning

Total RNA was extracted from the proximal intestine of sea bass using PureYield RNA Midiprep System (Promega), following the manufacturer's protocol. The extracted RNA was spectrophotometrically quantified using a NanoDrop instrument (Thermo Scientific) and its integrity was checked on agarose gel. After extraction, total RNA was reverse transcribed into cDNA in a reaction mix containing oligo dT primer, dNTPs and the Superscript III RT (Life Biotechnologies).

To perform PCR, a sample of the resulting cDNA was amplified using GoTaq Polymerase (Promega); the primer sets (forward and reverse) are listed in online Supplementary Table S1. To design the primers, a BlastN search (http://www.ncbi.nml.nih.gov/BLAST/) for orthologues of the *SLC6A19* gene in other fish/vertebrate species was performed. A multiple nucleotide sequence alignment (http://www.ebi.ac.uk/Tools/msa/clustalw2/) was then carried out with these sequences and three couples of primers were designed on regions of strong nucleotide conservation. The PCR amplifications were performed utilising an automated Thermal Cycler (Mycycler, Biorad). The PCR products were loaded on agarose gel and purified. The amplicons generated were then cloned in pGEM^®^-T Easy vector (Promega) and sequenced in both directions, T7 and SP6.

The full-length cDNA for *SLC6A19* was subsequently obtained by using the RACE method (rapid amplification of the cDNA ends). The 5′ and 3′ ends of the *SLC6A19* transcript were amplified using the 5′ RACE System kit and the 3′ RACE System kit (Life Technologies), respectively. The resulting products were run on a 2 % agarose gel, purified, cloned into the pGEM-T easy vector, and sequenced.

### Quantitative One-Step TaqMan® real-time PCR

#### Generation of *in vitro*-transcribed *SLC6A19* and *PEPT1* mRNA for standard curves

*SLC6A19* and *PEPT1* mRNA copies were absolutely quantified by one-step real-time PCR using the standard curve (absolute) method. A standard curve was constructed for every target gene using the known mRNA copy number of that gene. For this purpose, a forward and a reverse primer were designed based on the mRNA sequences of sea bass *SLC6A19* or *PEPT1* (see online Supplementary Table S1). Each forward primer was engineered to contain a sequence at its 5′ end corresponding to bacteriophage T7 RNA polymerase promoter. Forward and reverse primer pairs were used to create templates for the *in vitro* transcription of mRNA for *SLC6A19* and *PEPT1. In vitro* transcription was performed using T7 RNA polymerase and other reagents supplied in the Promega RiboProbe *In Vitro* Transcription System kit following the manufacturer's protocol. The molecular weight (MW) of the *in vitro*-transcribed RNA for each gene was calculated according to the following formula:

MW = (129 (no. of A bases) × 329·2 + 69 (no. of U bases) × 306·2 + 66 (no. of C bases) × 305·2 + 98 (no. of G bases) × 345·2 + 159).

The mRNA produced by *in vitro* transcription were then used as quantitative standards for analysing the experimental samples using one-step TaqMan^®^ EZ RT-PCR Core Reagents (Life Technologies). RT-PCR conditions were: 2 min at 50 °C, 30 min at 60 °C, and 5 min at 95 °C, followed by forty cycles consisting of 20 s at 92 °C and 1 min at 62 °C. The cycle threshold (Ct) values obtained by amplification were used to create standard curves for target genes.

### Quantification of *SLC6A19* and *PEPT1* mRNA copies by one-step TaqMan® real-time PCR

A quantity of 100 ng of total RNA extracted from the each experimental sample was subjected in parallel to 10-fold-diluted defined amounts of *in vitro*-transcribed mRNA to one-step real-time PCR (quantitative RT-PCR; qRT-PCR) under the same experimental conditions as used to establish the standard curves. Real-time Assays-by-Design custom primers and gene-specific TaqMan^®^ probes were designed by Life Technologies.

The following primer sequences and TaqMan^®^ probe of the cloned *SLC6A19* (accession no. KC812315) target gene were used:

Forward primer: 5′-TCACCTGTGTGGGCTTTTGT-3′;

Reverse primer: 5′-CCTTACCTGTGTCAAAGCCATG-3′;

TaqMan^®^ probe: 5′-GTGGGACTCGGCAACGT-3′.

The following primer sequences and TaqMan^®^ probe of the *PEPT1* (accession no. FJ237043) target gene were used:

Forward primer: 5′-GCTACCCTCTGGCCTTTGG-3′;

Reverse primer: 5′-ATGGTGGTAGCTCTGATTGTGTTC-3′;

TaqMan^®^ probe: 5′-TCCCCGCTGCTCTC-3′.

qRT-PCR was performed on a StepOne Real-Time PCR System (Life Technologies) and amplification data were collected by StepOne's Sequence Detector Program. The reaction efficiency was in the range 88–90 %. Furthermore, a minus-RT control (‘no amplification control’ or NAC) was included in qRT-PCR experiments. The NAC was a mock reverse transcription containing all the RT-PCR reagents, except the RT. No product was seen in the NAC, which indicates absence of DNA contamination.

### Calculation and statistical analysis

One-way ANOVA was used to determine whether there were any significant differences between the means of different groups. The level of statistical significance was set at *P* < 0·05.

### *In silico* analysis

The amino acid sequence of sea bass SLC6A19 transporter (GenBank accession number AGL33763) was analysed using the open-reading frame (ORF) finder software, which is freely available on the NCBI website (http://www.ncbi.nlm.nih.gov). The nucleotide sequence was compared with other sequences deposited in the GenBank protein database using the BLASTP algorithm^(^^38^^,^^39^^)^. Sequences were aligned using the ClustalW program^(^^40^^–^^42^^)^ and Multiple Sequence Alignments Editor & Shading Utility, GeneDoc, version 2.6.002 (http://www.nrbsc.org/downloads/).

### Protein annotation, tertiary structure and sites for post-translational modifications

Please see paragraphs in the online Supplementary material.

## Results

### Molecular cloning of sea bass *SLC6A19* cDNA sequence

A BlastN search was carried out (http://www.ncbi.nlm.nih.gov/BLAST/) in the complete, non-redundant GenBank nucleotide database for orthologues of *SLC6A19* in other fish species.

A multiple-sequence nucleotide alignment was then performed on coding sequences found for *SLC6A19*, and a strategy based on regions of strong nucleotide conservation was used to design the primers. Primer design was based on the multiple-sequence alignment of two teleost and one mammal *SLC6A19* cDNA coding sequences available in the GenBank database: *Oreochromis niloticus* (accession no. XM_003448888), *Salmo salar* (accession no. NM_001141815) and *Rattus novergicus* (accession no. EF474455). These presented several conserved regions within the sequence where primers could be reasonably designed. Three partially overlapping cDNA fragments were obtained by PCR using the designed primers. Then, by connecting the sequences of the partially overlapping clones, a partial coding sequence was determined for sea bass *SLC6A19*. The full-length cDNA for *SLC6A19* was subsequently isolated by 5′- and 3′-RACE and deposited in the GenBank database under the accession no. KC812315. The sea bass *SLC6A19* cDNA consists of 2110 bp, comprising a 5′-untranslated region (140 bp), an ORF (1905 bp) and a 3′-untranslated region (65 bp), including the possible polyadenylation signal (AATAAA).

The deduced amino acid sequence of sea bass *SLC6A19* (GenBank accession no. AGL33763) consisted of 634 amino acids with a deduced molecular mass of approximately 71·6 kDa.

### *In silico* analysis of sea bass SLC6A19 protein

**Fig. 1.**
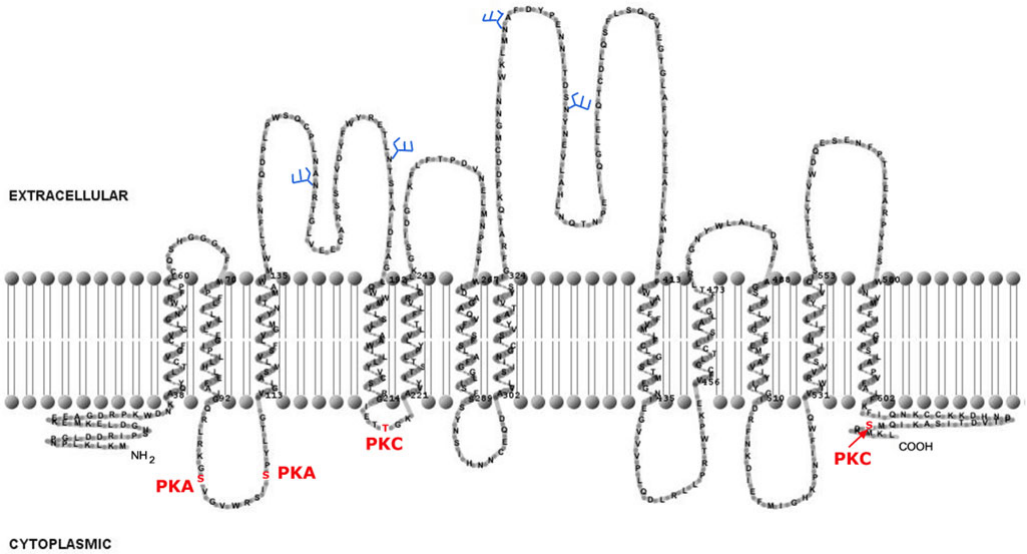
The twelve membrane-spanning helices in sea bass (*Dicentrarchus labrax*) solute carrier family 6 member 19 (SLC6A19) protein predicted with the TMHMM 2.0 program (http://www.cbs.dtu.dk/services/TMHMM/) and constructed with the TMRPres2D program. The amino acid residues are named in dark letters, and the N-glycosylation (blue branched) and phosphorylation sites (residues labelled in red) are highlighted. PKA, protein kinase A; PKC, protein kinase C.

The sea bass SLC6A19, predicted with both the HMM-TM and the TMHMM2.0 programs (http://www.cbs.dtu.dk/services/TMHMM/), adopts a twelve-transmembrane domain structure, with the amino- and carboxyl-terminus facing the cytosol and two large extracellular loops between the membrane-spanning helices (MSH) 3 and 4 and 7 and 8 (Fig. 1).

The similarity percentages for the alignment of SLC6A19 in different species, including the protein sizes, are presented in Table 2. Sea bass SLC6A19 showed the highest sequence identity with teleosts (Nile tilapia (*Oreochromis niloticus*), 88 %; Zebra mbuna (*Maylandia zebra*) and Japanese medaka (*Oryzias latipes*), 87 %; Japanese pufferfish (*Takifugu rubripes*) and zebrafish (*Danio rerio*), 75 %) and avian species (chicken (*Gallus gallus*) and turkey (*Meleagris gallopavo*), 72 %; zebra finch (*Taeniopygia guttata*), 71 %) and lower percentage identity with mammalian species (dog (*Canis lupus familiaris*), 72 %; rat (*Rattus norvegicus*), giant panda (*Ailuropoda melanoleuca*) and domestic rabbit (*Oryctolagus cuniculus*), 71 %; cattle (*Bos taurus*) and pig (*Sus scrofa*), 70 %; horse (*Equus caballus*), 69 %).

**Table 2.** Shared identities (%) between solute carrier family 6 member 19 (SLC6A19) proteins in different teleost, avian and mammalian species

Gene	Species	GenBank accession number	Protein size (aa)	Identity with *Dicentrarchus labrax* (%)
SLC6A19	*Dicentrarchus labrax*	AGL33763	634	–
	*Oreochromis niloticus*	XP_003448936	635	88
	*Maylandia zebra*	XP_004547827	635	87
	*Oryzias latipes*	XP_004074459	641	87
	*Takifugu rubripes*	XP_003969076	638	85
	*Danio rerio*	XP_002665492	647	75
	*Meleagris gallopavo*	XP_003204921	636	72
	*Rattus norvegicus*	ABP63542	634	71
	*Gallus gallus*	XP_419056	636	72
	*Taeniopygia guttata*	XP_002187651	635	71
	*Canis lupus familiaris*	XP_003434149	634	72
	*Sus scrofa*	XP_003359903	634	70
	*Bos taurus*	XP_003583760	634	70
	*Ailuropoda melanoleuca*	XP_002918877	634	71
	*Oryctolagus cuniculus*	NP_001156588	634	71
	*Equus caballus*	XP_001499282	634	69

aa, Amino acids.

### Protein annotation, sites for post-translational modifications and tertiary structure

Please see the online Supplementary material.

### Effects of the diets on growth performance

Table 3 summarises the growth rate and final feed conversion ratio (FCR) achieved by the fish in response to different diets. Survival was high (about 95 %) with no significant differences among the fish groups fed different diets. The lowest daily feed consumption was obtained in fish that received diet no. 5 (10 % FM and no salt added) in comparison with all other groups. Fish fed this diet also exhibited the lowest SGR, and the poorest FCR. In comparison, fish that were fed diet no. 6, which contained 10 % FM and 3 % NaCl, showed significantly enhanced feed intake and SGR and FCR levels. Moreover, no significant differences in the SGR or FCR values were observed between fish fed diet no. 4 (30 % FM inclusion) and diet no. 6, suggesting that the addition of salt in the diet compensated for the role of FM as a source of NaCl.

**Table 3.** Weight gain and feed conversion ratio (FCR) of juvenile sea bass (*Dicentrarchus labrax*) at the end of the 6-week growth trial (Mean values with their pooled standard errors)

	Diet 1 (HFM/LF)	Diet 2 (HFM/MF)	Diet 3 (HFM/HF)	Diet 4 (MFM/LF)	Diet 5 (LFM/LF)	Diet 6 (LFM/LF + NaCl)	Pooled sem
Specific growth rate (%)†	2·06^a^	1·99^a^	2·10^a^	1·51^b^	0·89^c^	1·40^b^	0·043
FCR‡	1·20^a^	1·21^a^	1·21^a^	1·57^b^	2·66^c^	1·72^b^	0·062
Total feed intake (g/fish)	17·54^a,b^	18·07^a^	18·72^a^	14·73^c^	13·41^c^	15·89^b^	0·053

HFM, high fish meal (50 %); LF, low fat (16 %); MF, medium fat (18 %); HF, high fat (20 %); MFM, medium fish meal (30 %); LFM, low fish meal (10 %).

^a,b,c^ Mean values within a row with unlike superscript letters were significantly different (*P* < 0·05).

* The data were tested by ANOVA followed by Tukey's honest significant difference (HSD) test.

† Specific growth rate was calculated using the following formula: (ln W_f_ – ln W_i_)/t x 100, where W_f_ is the final weight (g), W_i_ is the initial weight (g) and t is growth time (d).

‡ FCR = the amount of feed (in g/kg) given or required in order to obtain the measured weight gain. FCR is a measure of an animal's efficiency in converting feed mass into increases of the desired output.

There were no significant differences in SGR, FCR and total feed intake among the fish fed diets no. 1, 2 and 3, which contained the same percentage of FM but increasing levels of fat.

### Spatial distribution of *SLC6A19* mRNA in sea bass digestive tract

Total RNA extracted from sea bass gut sections was subjected to one-step real-time RT-PCR using the standard curve method to determine absolute amounts of *SLC6A19* mRNA. The standard curve created for *SLC6A19* was based on the linear relationship between the Ct value and the logarithm of the starting amount. To obtain Ct values corresponding to defined transcript copies for the target gene, defined quantities at 10-fold dilutions of *SLC6A19* mRNA were run together with the samples in a forty-eight-well real-time plate. The gut sections analysed were evaluated using repeated real-time quantifications taken in spatial sequence as described by Littell *et al.*^(^^43^^)^.

**Fig. 2.**
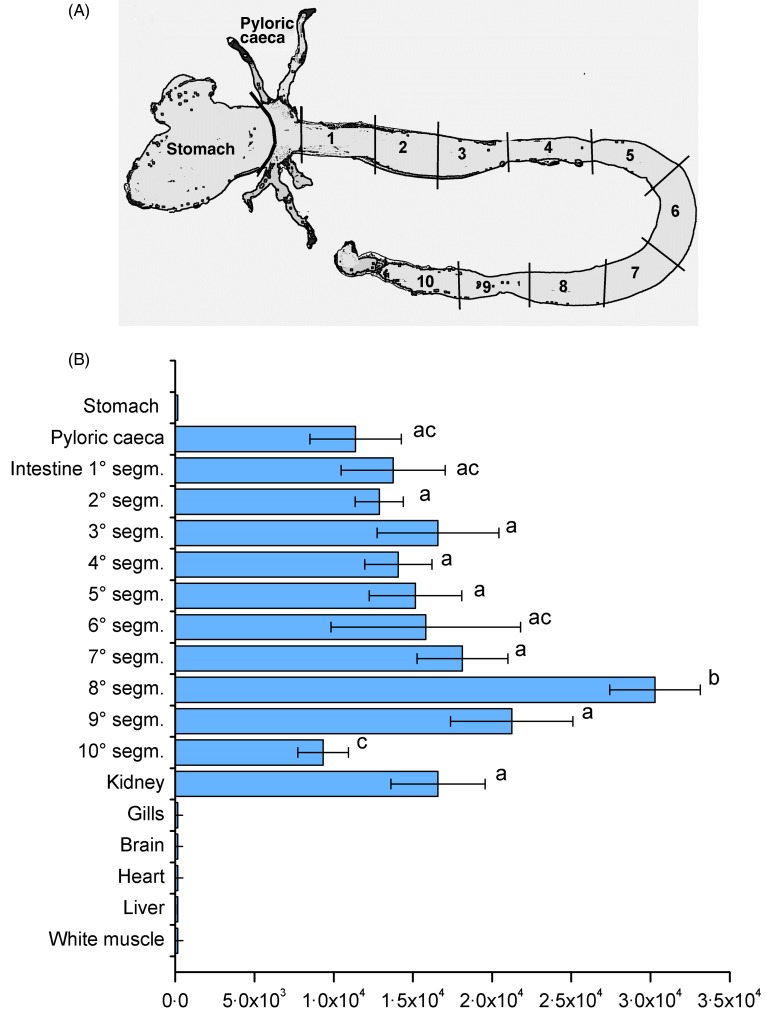
Spatial distribution of sea bass (*Dicentrarchus labrax*) solute carrier family 6 member 19 (*SLC6A19*) mRNA along the digestive tract and in other organs/tissues as determined by real-time quantitative PCR. (A) Picture of sea bass digestive tract: stomach, pyloric caeca and ten adjacent intestinal segments (segm.) starting after the pyloric caeca area. (B) Expression levels of *SLC6A19* measured by real-time PCR in the different segments of the sea bass digestive tract. *SLC6A19* mRNA copy number was normalised as a ratio to ng total RNA. Values are means of five animals per group, with standard errors represented by horizontal bars. ^a,b,c^ Mean values with unlike letters were significantly different (*P* < 0·05).

This analysis revealed the following spatial distribution of *SLC6A19* transcripts in the sea bass digestive tract (Fig. 2(A) and (B)): very high levels of expression in segments 8 and 9 of the intestine, high levels in intestinal segments 1–7, and lower levels in the most distal part of the intestine (segment 10) and in the stomach. Such distribution might indicate specific functions of different regions in the intestinal tract of sea bass and could be related to functional differences in the amino acid-absorbing capacity of the gut.

### Quantification of *SLC6A19* and *PEPT1* mRNA copy number in sea bass proximal and distal intestine in response to different diets

**Fig. 3.**
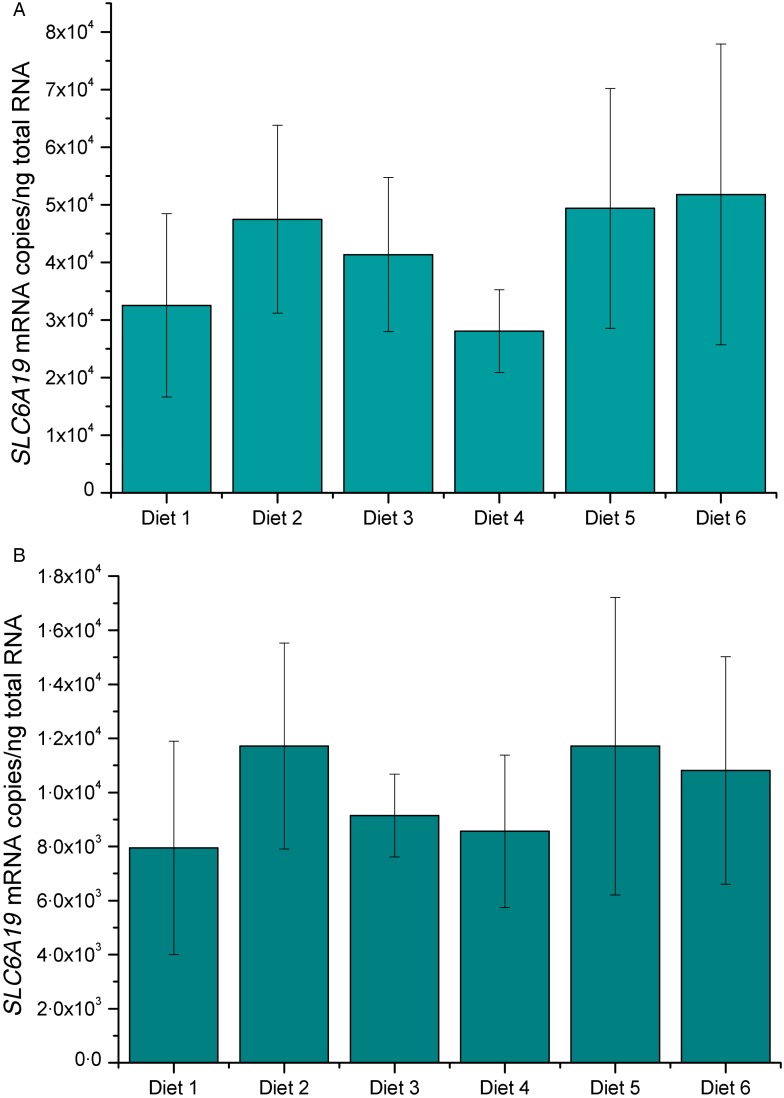
Expression levels of sea bass (*Dicentrarchus labrax*) solute carrier family 6 member 19 (*SLC6A19*) measured by real-time PCR in hindgut (A) and foregut (B) at the end of the experiment. Fish were fed six different diets: diet 1 (50 % fish meal (FM)/16 % fat); diet 2 (50 % FM/18 % fat); diet 3 (50 % FM/20 % fat); diet 4 (30 % FM/16 % fat); diet 5 (10 % FM/16 % fat); diet 6 (10 % FM/16 % fat + NaCl). *SLC6A19* mRNA copy number was normalised as a ratio to ng total RNA. Values are means of five animals per group, with standard errors represented by vertical bars. One-way ANOVA indicated that there were no significant differences. The level of statistical significance was set at *P* < 0·05.

The absolute mRNA levels of *SLC6A19* in the proximal (segments 1–3) and distal (segments 9–10) intestine of sea bass in response to the feeding trial are presented in Fig. 3(A) and (B). It can be seen that fish fed *ad libitum* for 6 weeks with six different diets (45 % protein, 16–20 % fat, from 40 to 80 % of the FM replacement) did not show significantly different levels of *SLC6A19* transcripts (*P* < 0·05) in the foregut (Fig. 3(A)) as compared with the control group (diet no. 1; 50 % FM and 16 % fat). *SLC6A19* mRNA copies in the hindgut of fish fed the six diets were also not significantly different from the controls (Fig. 3(B)).

**Fig. 4.**
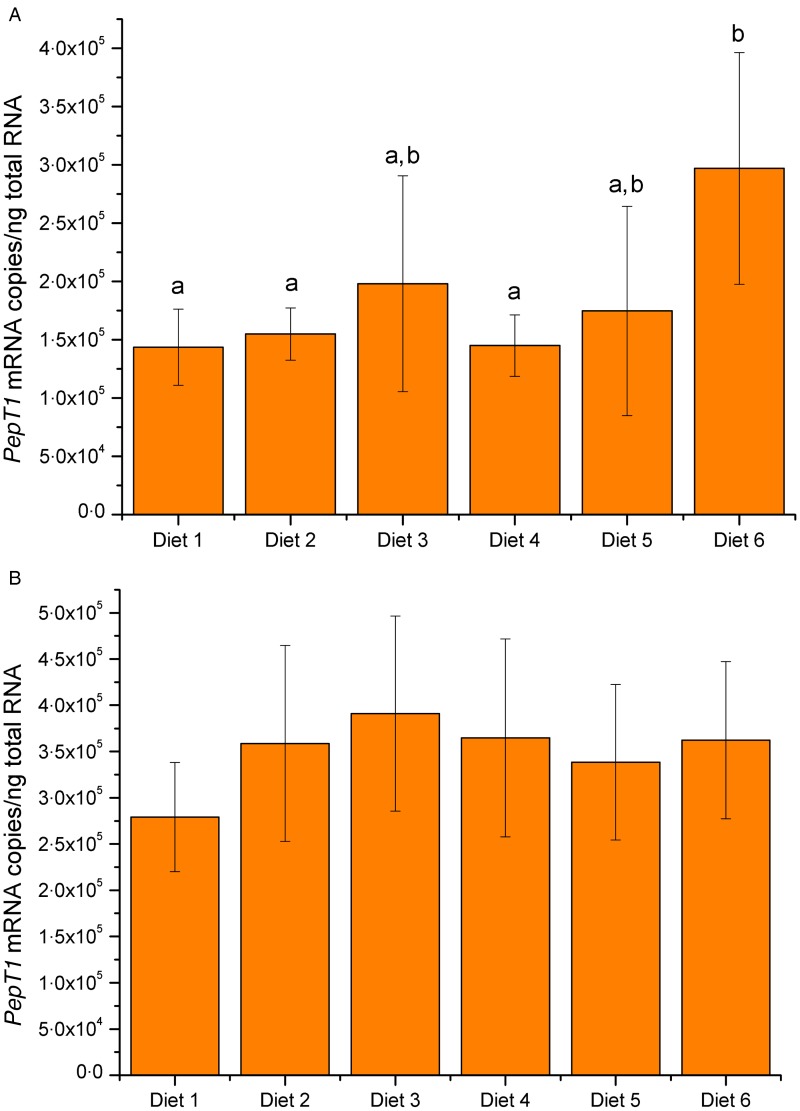
Expression levels of oligopeptide transporter 1 (*PepT1*) gene measured by real-time PCR in hindgut (A) and foregut (B) of sea bass (*Dicentrarchus labrax*) at the end of the experiment. Fish were fed six different diets: diet 1 (50 % fish meal (FM)/16 % fat); diet 2 (50 % FM/18 % fat); diet 3 (50 % FM/20 % fat); diet 4 (30 % FM/16 % fat); diet 5 (10 % FM/16 % fat); diet 6 (10 % FM/16 % fat + NaCl). *PepT1* mRNA copy number was normalised as a ratio to ng total RNA. Values are means of five animals per group, with standard errors represented by vertical bars. ^a,b^ Mean values with unlike letters were significantly different (*P* < 0·05; one-way ANOVA).

Unlike *SLC6A19, PEPT1* gene expression levels in the hindgut of sea bass (Fig. 4(A)) were significantly higher in the fish fed diet no. 6 (10 % FM, 16 % fat and 3 % NaCl) than in fish fed diets no. 1 (50 % FM and 16 % fat), no. 2 (50 % FM and 18 % fat) and no. 4 (30 % FM and 16 % fat), with fish fed diet no. 1 presenting the lowest mRNA copies of *PEPT1*. Fish fed diets no. 3 (50 % FM and 20 % fat) and no. 5 (10 % FM and 16 % fat) showed intermediate levels of the dipeptide transporter mRNA copies, which did not differ significantly either from fish fed diet no. 6 or those fed diets no. 1, no. 2 and no. 4. The copy number of *PEPT1* mRNA in the foregut of sea bass was not influenced by different diets, as shown in the lower panel of Fig. 4(B).

## Discussion

In the present study, a full-length transcript encoding for the intestinal neutral amino acid transporter *SLC6A19* was successfully identified in sea bass.

The ‘*in silico*’-deduced protein showed a good alignment with sequences of other teleost SLC6 transporters available in the Swiss-Prot database. Sea bass *SLC6A19* exhibited a high sequence identity with orthologues in other teleosts; otherwise, this transporter is well conserved in all classes of vertebrates.

The main result of our bioinformatics analysis on the amino acid sequence is the ‘*de novo*’ prediction of the tertiary structure of sea bass SLC6A19 (see online Supplementary material), demonstrating similarities with the LeuT of *Aquifex aeolicus*, which is considered to be the first crystal structure of a homologue of the neurotransmitter/sodium symporter deposited in the Protein Data Bank (PDB) (ID: 2A65).

The SLC6A19 putative protein of *D. labrax* was correctly predicted to be an integral membrane symporter belonging to the solute carrier family 6 (SLC6), which is characterised by twelve transmembrane domains with the N- and C-terminus inside the cytosol and two elongated extracellular loops connecting the third and fourth and the seventh and eighth transmembrane segments, respectively. The extended length of the second and fourth extracellular loops is characteristic of SLC6A protein transporters known as orphan transporters because they are not completely characterised and grouped in a distinctive cluster of the four-branched tree describing the SLC6 protein family^(^^10^^,^^44^^)^. These two long loops host all the N-glycosylation sites: two on the second and two on the fourth loop (Fig. 1). Similar glycosylated loops have been reported by Takanaga *et al.*^(^^44^^)^ in human SLC6A15 (also called B^0^AT2), which has a 35–40 % identity with the human epithelial transporter SLC6A19.

N-glycosylation constitutes an important post-translational modification in members of the SLC6 family because it plays a role in uptake activity^(^^45^^)^. All four N-glycosylation sites in sea bass SLC6A19 were predicted to be accessible: the ‘*in silico*’ simulation of their binding to oligomannose (see online Supplementary material) showed that all the sugars are placed at the extracellular surface, around the port of entry to the active pocket. Conversely, C- or O-glycosylation was not allowed in sea bass SLC6A19. The putative protein contained no signal peptide or glycosylphosphatidylinositol (GPI) anchor^(^^46^^)^; however, it should be kept in mind that the server utilised for the analysis was based on a mammal protein database and was thus, realistically, less reliable for study of lower vertebrate proteins.

Phosphorylation is another post-translational modification usually found in this family of proteins and a number of phosphorylation sites were predicted for sea bass SLC6A19. Those reported by Takanaga *et al.*^(^^44^^)^ in human subjects were used as a reference for comparison in our analysis (see online Supplementary material). The similarity was evident but, despite the high score obtained, two phosphorylation sites were not considered (T532 and S386) because they were found in a poorly accessible region, i.e. along a helix and on an extracellular loop, respectively.

The key objective of the present study, however, was to investigate the effects of different diets on the expression of intestinal transporter genes *SLC6A19* and *PEPT1* in sea bass. Over the past two decades, partial or total replacement of FM in the diets for most cultivated fish species has been the subject of numerous studies. Despite some variability between and within fish species, the large majority of studies performed with reputedly ‘carnivorous’ finfish tend to show that a partial (30 % to 40 %) replacement of dietary FM protein by a single plant-protein source can be achieved but that at higher inclusion levels, the overall growth performance and feed utilisation tend to be depressed^(^^47^^)^. Dias *et al.*^(^^48^^)^ showed in their study that a high replacement level (up to 80 %) of FM by a single plant-protein source such as soya protein concentrates or maize gluten meal had an adverse effect on overall growth performance and protein or energy utilisation in European sea bass. Previous studies on this species had also reported reduced growth and lower protein efficiency ratios when the levels of soya proteins or maize gluten meal exceeded 20–30 %^(^^49^^,^^50^^)^. Otherwise, extruded diets containing more than 25 % FM and different levels of legumes showed no significant difference in the growth performance of sea bass^(^^51^^)^. In the present study, we used six isonitrogenous diets (45 % protein) with either 40 or 80 % of the FM replaced by defatted soyabean and soya protein concentrate.

As expected, the present results confirmed that the FM content in the diets was positively correlated with the SGR and FCR of juvenile European sea bass. High dietary levels (50 %) of FM attained the best growth performance, whereas the SGR of sea bass fed a diet containing 10 % FM was significantly lower. Of greater interest is the fact that the addition of 3 % salt to the diet poorest in FM (containing only 10 % FM) resulted in improved growth parameters, which became similar to those observed in fish fed the diet containing 30 % FM. Indeed, dietary salt supplementation improved feed uptake, SGR and FCR, which reached the same values as those in fish fed a diet containing 30 % FM. These results are consistent with other studies, which have shown that dietary salt supplementation has a positive effect on fish growth performance under freshwater conditions^(^^36^^,^^52^^,^^53^^)^. Adding salt to the diet of Asian sea bass at a level of up to 4 % led to a better feed utilisation^(^^53^^)^. A diet without FM but supplemented with 3 % NaCl significantly increased the SGR and FCR of a tilapia hybrid^(^^52^^)^. It is known that the regulatory mechanism of dietary salt supplementation involves digestive enzyme activities in the pyloric caeca and plasma osmolality^(^^53^^)^. Sun *et al.*^(^^54^^)^ recently demonstrated that adding dietary salt also affects the intestinal bacterial community residing in sea bass. In their study, supplementation of 3 % NaCl to a diet containing 10 % FM resulted in a bacterial community that was more similar to that found in fish fed diets with higher levels of FM. The significance of this finding lies in the fact that the composition of the microbial community in the gut clearly affects the amount of energy extracted from the diet, which is ultimately related to weight gain^(^^55^^)^.

The increased use of plant ingredients in aquafeeds can produce inflammatory reactions in the intestine, compromising nutrient uptake and, hence, fish health and welfare^(^^56^^,^^57^^)^. Nutrient uptake from the intestinal lumen involves different transport proteins that bind and move nutrients into the cells across the brush-border membrane. Several methods have been used to determine diet digestibility in aquaculture species but only a few studies tried to correlate feed absorption with the expression levels of intestinal amino acid and oligopeptide transporters^(^^33^^,^^58^^,^^59^^)^. Knowledge about intestinal transporter expression is important because transport regulation may be used as a means of increasing the growth rate.

In the present work, we used real-time PCR methodology to investigate the effects of high dietary levels of soyabean proteins on the expression of SLC6A19 and PEPT1 transporters in the intestinal tract of sea bass.

Similarly to mammal orthologues, the neutral amino acid transporter SLC6A19 is abundantly expressed in the intestine, pyloric caeca and kidney of sea bass, but is also expressed at low levels in the most distal part of the intestine. PEPT1, instead, is mainly expressed in the proximal intestine and pyloric caeca of this species, but it is also expressed at very low levels in the stomach and in the distal part of the intestine^(^^28^^)^.

The results of the present study clearly indicate that expression of the *Slc619* gene was not affected by different soyabean-based diets. The lack of significant differences in SLC6A19 expression levels in response to different diets may be explained by the fact that free amino acids are transported not only by SLC6A19, but also by other amino acid transporters, which vary in substrate specificity and exhibit various ion dependencies and mechanisms for translocating amino acids^(^^12^^)^. Conversely, di- and tri-peptides are transported into the enterocytes exclusively by the peptide transporter PEPT1 and, if transport of neutral amino acids in the small intestine fails, PEPT1 can compensate for the reduced amino acid delivery; indeed, both dipeptides and free amino acids induce *PEPT1* expression in the fish intestine^(^^59^^)^. In the present study the intestinal expression of *PEPT1* was affected by diet composition, even though only in sea bass hindgut. This is in agreement with what we have previously observed in another marine fish species, the gilthead sea bream. Indeed, in sea bream *PEPT1* mRNA levels in intestine were influenced by different feeds in which various vegetable sources were substituted for the FM^(^^33^^)^. In this study, *PEPT1* mRNA levels were found to be down-regulated in the proximal intestine of fish fed a diet containing 15 % green pea meal and growth was lower than in the controls, demonstrating that *PEPT1* expression levels were related to fish performance during the feeding trial. Several other studies have suggested that up-regulated *PEPT1* expression by diet in teleosts could stimulate growth rate directly or indirectly^(^^31^^,^^58^^,^^59^^)^. Surprisingly, in the present work, a higher expression level of the *PEPT1* gene was observed in those fish fed the diet with the lowest FM content (10 %) but with salt added. In the same fish group we found good SGR values, i.e. a SGR comparable with those fish fed a 30 % FM diet. Conversely, fish receiving the same diet (10 % FM) but without salt supplementation showed the lowest SGR value and low *PEPT1* expression. The positive effect of dietary salt supplementation on fish growth performance, with respect to the diet without salt, might be attributed to an improvement in sea bass intestinal transporter activity, in particular of SLC6A19 protein activity, as in sea bass, like in mammals, it was demonstrated that SLC6A19 is strongly Na^+^ dependent^(^^17^^)^.

In conclusion, we isolated a cDNA sequence encoding for the neutral amino acid transporter *SLC6A19* in sea bass (*Dicentrarchus labrax*) and characterised the three-dimensional structure of the protein. We then quantified by one-step TaqMan real-time PCR the mRNA copies of *SLC6A19* and oligopeptide transporter, *PEPT1*, in different intestinal portions of fish fed on different diets. *SLC6A19* mRNA copies in the anterior and posterior intestine of freshwater-adapted sea bass were not modulated by dietary protein sources and salt supplementation. Conversely, including salt in a diet containing a low FM percentage up-regulated the mRNA copies of *PEPT1* in seabass hindgut. The beneficial action of dietary salt supplementation on fish growth performance, in a diet containing a low FM percentage, might suggest that Na^+^ ions could be traced to an improvement in the activity of intestinal transporters, in particular of the Na^+^-dependent neutral amino acid transporter SLC6A19.

## Supplementary material

For supplementary material accompanying this paper visit http://dx.doi.org/10.1017/jns.2015.9. click here to view supplementary material
