# Supplementary material for: Intestinal B0AT1 (SLC6A19) and PEPT1 (SLC15A1) mRNA levels in European sea bass (Dicentrarchus labrax) reared in fresh water and fed fish and plant protein sources
Source: J Nutr Sci. 2015 May 20;4:e21. doi: 10.1017/jns.2015.9 (PMC4462763; doi:10.1017/jns.2015.9)
Supplement: Supplementary file 1 [file S2048679015000099sup001.doc]

Online Supportive Material

**Materials and Methods**

**Table S1** Sequences of the primers used in the work

| **Primer** | **Sequence 5’ – 3’** | **Purpose** |
| --- | --- | --- |
| SLC6A19-fw1 | GGCCCAAATGGGACAACAA | RT-PCR |
| SLC6A19-rv1 | CAGAGTTGTAGCTGGAGAAGGAG | RT-PCR |
| SLC6A19-fw2 | TGGATGCAGGTGCTCAAGT | RT-PCR |
| SLC6A19-rv2 | CCAGACAGAAGAGCATGATGAA | RT-PCR/mRNA std.curve |
| SLC6A19-fw3 / 3’GSP1 | TGCCTGTTTCTCCTCTCTGG | RT-PCR/3’ RACE |
| SLC6A19-rv3 | GACTTTGGTGACGAAGTAGAA | RT-PCR |
| SLC6A19-T7fw | *gtaatacgactcactataggg*GGCCCAAATGGGACAACAA | mRNA standard curve |
| 5’GSP1 | TGTCCTGTTAGCATTGAGAG | 5’ RACE |
| 5’GSP2 | CCTTTCCTCAGTCGCTGTCC | 5’ RACE |
| 5’GSP3 | ATGCTCCTCCTCCATGGCTT | 5’ RACE |
| 3’GSP2 | TCTGGTCATCGGATTCTGTG | 3’ RACE |
| PepT1-T7fw | gtaatacgactcactatagggGGAATGTGGCATTCACACC | mRNA std. curve |
| PepT1-rv | AGCAGGGCTCAAGATGGAC | mRNA std. curve |

***In silico* analysis**

***Protein annotation***

The putative amino acid sequence of sea bass was compared with the complete orthologous sequences of three teleosts: Atlantic salmon (*Salmo salar*) (accession no. **NM_001141815**), Nile tilapia (*Oreochromis niloticus*) (accession no. **XM_003448888**), and zebrafish (*Danio rerio*) (accession no. **BC059804**).

Although a high degree of similarity was found for the protein sequence of sea bass SLC6A19 to its orthologous counterparts, no information was available on protein annotation; therefore, an analysis was performed on the primary structure of sea bass SLC6A19 protein.

The presence of conserved domains and patterns inside the putative amino acid sequence was checked through different programs: PROSITE (1), SMART 7 (2, 3), InterPro (4), and CDD (5, 6, 7).

***Tertiary structure***

The protein sequence of sea bass SLC6A19 aligns partially with only one X-ray-solved structure present in the Protein Database (PDB; entry: 2A65; E-value: 2e-22) (http://www.pdb.org/pdb/home/home.do).

As the alignment seemed to be poor, its tertiary structure could not be inferred from that in the template, and a “*de novo*” prediction of the tertiary structure of the putative protein was performed at the I-Tasser server. The four stages of the method implement a threading procedure followed by structural assembly, refinement of the model, and structure-based functional annotation. The output consists of five models, whose accuracy indexes are the C score, the TM score, and the RMSD (8, 9 10).

***Sites for post-translational modifications***

A) *Phosphorylation and sumoylation*

The serine, threonine, and tyrosine phosphorylation sites were searched using the NetPhos 2.0 server (11); sites for sumoylation were checked with the SUMOsp 2.0 program (12).

B) *Glycosylation*

The glycosylation sites present in the proteins were searched using the server of the CBS (Center for Biological Sequence Analysis, at the Technical University of Denmark, (http://www.cbs.dtu.dk/services/). Sites for mucin-type mannosyl-O-glycosylation (Net-O-glyc 4.0) (13), C-glycosylation (14), and ε-glycosylation of lysine residues (15) were checked and searched. When sites for N-glycosylation were predicted by the CBS (Net-N-glyc 1.0) (11, 16), their tertiary structure was described by using the GlyProt application of the Glycosciences server (http://www.glycosciences.de/modeling/).

**RESULTS**

**Protein annotation**

The SLC6A19 putative protein of *D. labrax* was classified according to the Gene Ontology terms retrieved by the InterPro (4) and the I-Tasser server. The protein belongs to the amino acid (nutrient) transporters (NATs) subfamily, one of the four branches of the SLC6 family. The protein is integral to the membrane (GO:0016021) and its main molecular function belongs to transporters of substances across the cell membrane (GO0005328). The biological process best describing this function is neurotransmitter transport (GO: 0006836). The amino acid transport scored lower (GO: 0003333).

The protein contains a conserved domain extending from positions 31 and 607 leading to the sodium neurotransmitter symporter (NSF) family profile. PROSITE (1) as well as SMART 7 (2, 3) and InterPro (4) recognized the domain (accession number: PS50267, PFAM: SNF and PTHR11616, respectively).

Both the HMM-TM program (17) and TMHMM2.0 (18, 19) similarly predicted the length and the topology of the helices (Fig. 1). The sea bass SLC6A19 adopted a 12 transmembrane domain structure, with the amino- and carboxyl-terminus facing the cytosol and two large extracellular loops between the membrane-spanning helices (MSH) 3 and 4 and 7 and 8 (Fig. 1).

**Sites for post-translational modifications**

Seven sites for lysine ε-glycosylation were predicted at positions 2, 4, 22, 331, 342, 454, and 631. Although no signal peptide or GPI anchor was present (20) and suitable sites for C- or O-glycosylation were correspondingly absent in the putative protein, five putative extracellular N-glycosylation sites (Asn^157^, Asn^181^, Asn^263^, Asn^353^, Asn^367^, ) were identified, and all but one were predicted as accessible at the Glycoscience server (<http://www.glycosciences.de/modeling/glyprot/php/main.php>)-. They are highlighted with blue-branched vignettes in Supplemental Fig. 1. The nonaccessible site, Asn^157^, fell inside the third transmembrane helix.

Four sites of possible sumoylation are present at amino acid positions K22, K25, K611, and K633. The SLC6A19 sequence contained several sites of phosphorylation along the entire chain (Ser: 52%, Thr: 28%, Tyr: 19%); four of them were also identified as putative binding sites for the intracellular cAMP/cGMP-dependent protein kinase A (Ser^99^ and Ser^107^) and C (Thr^218^ and Ser^629^) . All the features are highlighted along the primary structure in Supplemental Fig. 1.

**Tertiary structure**

The tertiary structure of the SLC6A19 protein of sea bass could not be inferred because of a low similarity with the solved structures retrieved from the PDB. Therefore, the main result in our bioinformatics analysis remained the “*de novo*” tertiary structure prediction obtained at the I-Tasser server v.2.0 (Supplemental Fig. 2).

We performed bioinformatics analyses with the first model obtained utilizing the I-TASSER server due to its higher values of C-score and TM-score in comparison with the other four models generated by I-TASSER. C score is a confidence score for estimating the quality of predicted models by I-TASSER. It is calculated according to the significance of threading template alignments and the convergence parameters of the structure assembly simulations. The C score is typically in the range of minus 5 to 2, where a higher C score signifies a model with a high confidence and vice versa. Both the TM score and RMSD are known standards for measuring structural similarity between two structures and usually used to measure the accuracy of structure modeling when the native structure is known.

SLC6A19 protein demonstrated similarities with the LeuTAq (Leucine transporter) from *Aquifex aeolicus*, considered to be the first crystal structure of a homologue of the neurotransmitter/sodium symporter and deposited in the PDB (ID: 2A65). The LeuT protein revealed an occluded binding pocket containing leucine and 2 Na^+^.

Accordingly, the presence of a functional channel similar to that described for the LeuT was confirmed by the COFACTOR tool (Supplemental Fig. 4). The binding site for sodium ions and leucine was predicted according to the crystallographically solved LeuTAq (Leucine transporter) (21), considered to be the first crystal structure of a homologue of the neurotransmitter/sodium symporter and deposited in the PDB (ID: 2A65). The four accessible sites for N-glycosylation, reconstructed “*in* *silico*” with the addition of oligomannose, are also highlighted in Supplemental Fig. 3.

MKLKLPNPGLDDRIPSHGDLEKMEKEEAGDRPKWDNKAQYLLTCVGFCVGLGNVWRFPYLCQSHGGGAFMIPFLILLVLE 80

GIPLLHLEFAIGQRLRKGSVGVWRSISPYLTGVGIASMLVSFLVGMYYNTIMAWIMWYLFNSFQDPLPWSQCPLNANRTG 160

LVEECARSSTVDYFWYRETLNTSTAIDEAGGLQWWMVLSLVAAWILLYVCSIRGIETTGKAVYITSTLPYLVLTIFLVRG 240

LTLKGSIDGIKFLFTPDVNELMNPSTWLDAGAQVFYSFSLAFGGLISFSSYNSIHNNCEQDAVLISIINGCTSVYSATVI 320

YSIIGFRATQKFDDCMGNNIWKLMNAFDYPENNITDSNYNEVLAHLNQTNPEIIQGLELQTCDLQSFLSQGVEGTGLAFI 400

VFTEAIIKMPVSPLWAVLFFVMLFCLGLSTMFGNIEGVVVPLQDLRLLPRTWPKEVFCGLTCLISLALGLIFTLRSGNYW 480

LALFDNFAGSIPLLVIGFCEMFAVIYIYGIDRFNKDIEFMIGHKPNIFWQVTWRVVSPLIMIFILIFYFVTQISKSLTYL 560

VWDQESENFPTLEARPYPSWINVMIFILAGVPSLAIPVFALFKFIQNKCCKKDHNDFTVDTISAKIQMSDKMKL 640

**Fig. S1** Summary of all the predicted sites of post-translational modification found in SLC6A19 of *D. labrax*, shown on the primary sequence. Color code: phosphorylation indicated as: S= blue, T= green, Y= red; glycosylation: K= yellow, N= pink. Sumoylation sites are underlined.


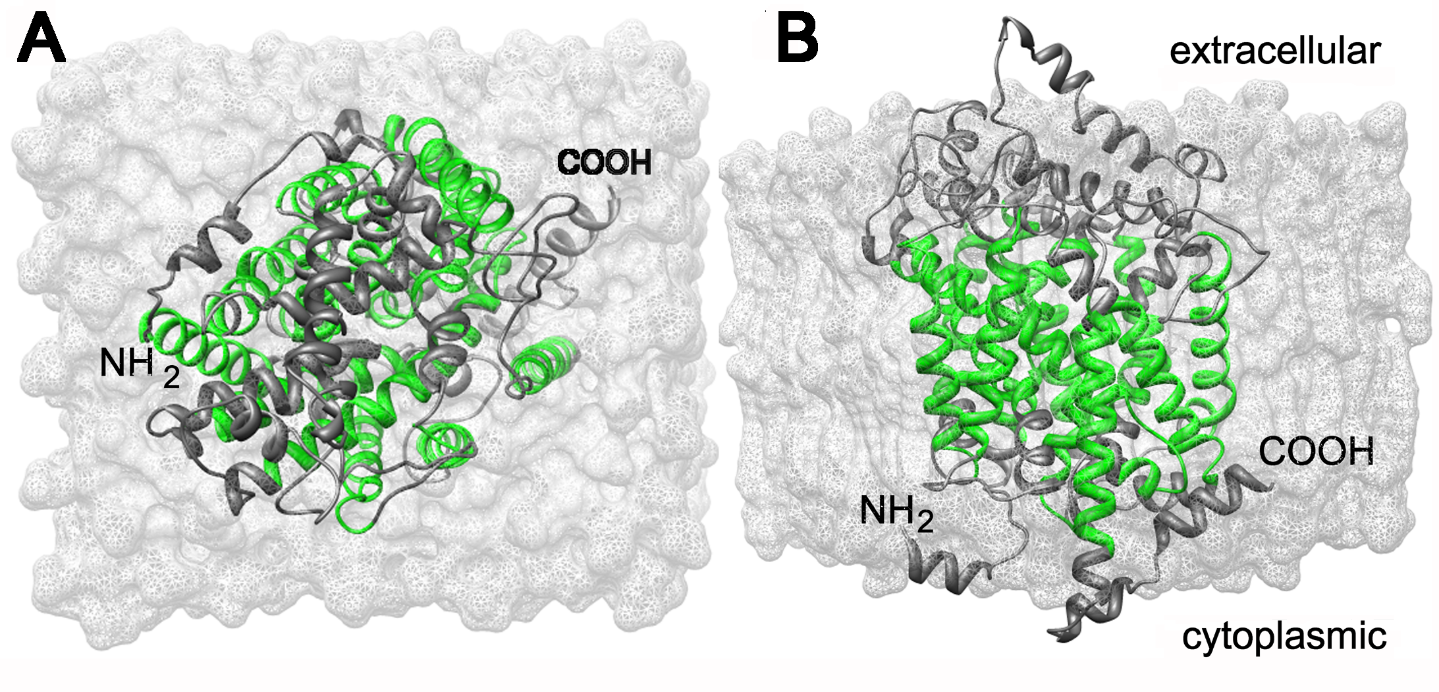


Fig. S2 Two prospects for the predicted 3D structure of the SLC6A19 of *D. labrax*: the extracellular surface is depicted in panel A, the lateral view in panel B. The transmembrane domains are highlighted in green.


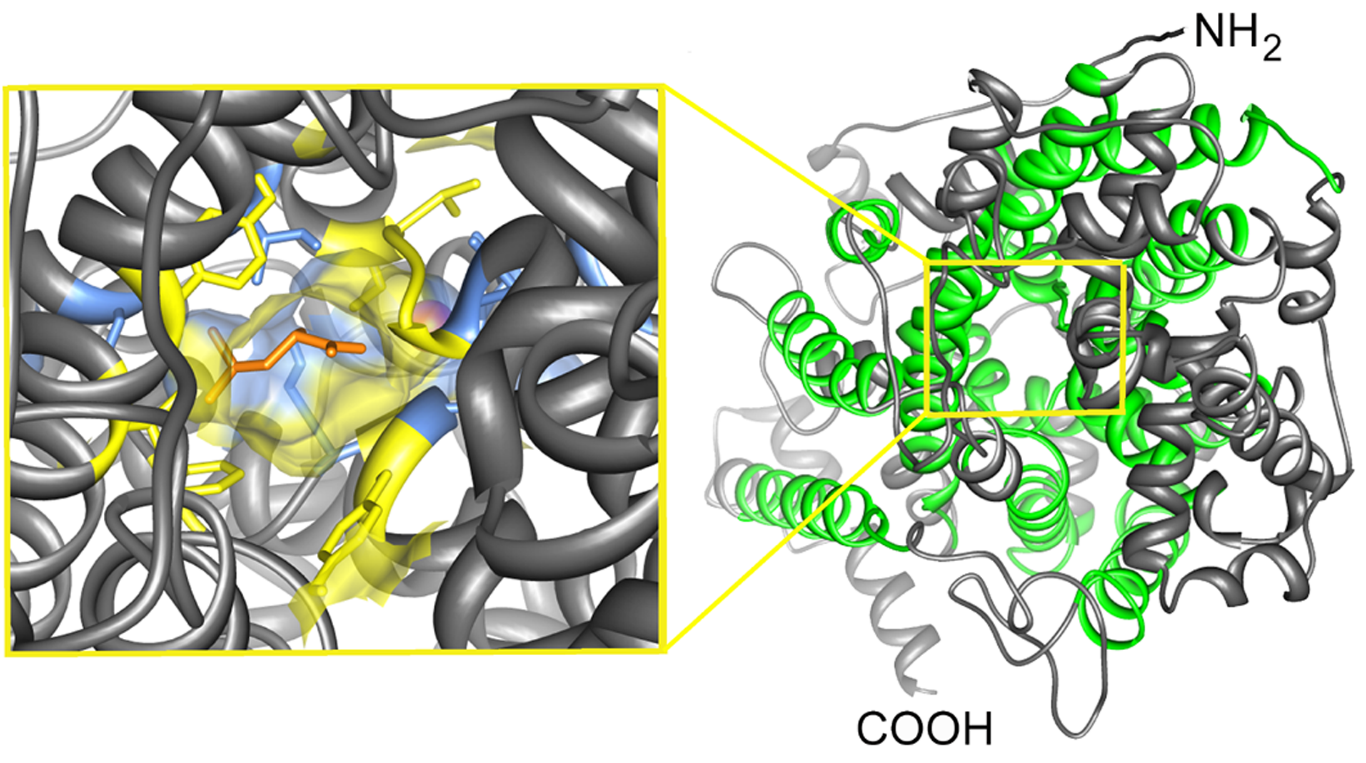


Fig. S3: The extracellular surface of SLC6A19 of *D. labrax* (right panel) and the functional channel, zoomed in the left panel. Color code: N-glycosylation accessible sites in blue, amino acids binding Na in yellow, and the leucine binding site in orange. The Na is a partially covered, red ball.

**References**

1. Sigrist CJA, Cerutti L, Hulo N, *et al.* (2002) PROSITE: a documented database using patterns and profiles as motif descriptors. *Brief Bioinform* **3**, 265–274 (<http://prosite.expasy.org/>).

2. Letunic I, Doerks T & Bork P (2012) SMART 7: recent updates to the protein domain annotation resource. *Nucleic Acids Res Jan* **40** (Database issue):D302–305. doi: 10.1093/nar/gkr931. (<http://smart.embl-heidelberg.de/>).

3. Schultz J, Milpetz F, Bork M, *et al.* (1998) SMART, a simple modular architecture research tool: Identification of signaling domains. *Proc Natl Acad Sci USA* **95**, 5857–5864 (<http://smart.embl-heidelberg.de/>).

4. Hunter S, Jones P, Mitchell A, *et al.* (2011) InterPro in 2011: new developments in the family and domain prediction database. *Nucleic Acids Res* **40** (Database issue):D306–312. doi:10.1093/nar/gkr948 (<http://www.ebi.ac.uk/interpro/>)

5. Marchler-Bauer A & Bryant SH (2004) CD-Search: protein domain annotations on the fly. *Nucleic Acids Research* **32**, Web Server issue W327–W331. (http://www.ncbi .nlm.nih.gov/cdd).

6. Marchler-Bauer A, Anderson JB, Chitsaz F, *et al.* (2009) CDD: specific functional annotation with the Conserved Domain Database. *Nucleic Acids Res* **37** (Database issue):D205-210.

7. Marchler-Bauer A, Zheng C, Chitsaz F, *et al.* (2013) CDD: conserved domains and protein three-dimensional structure. *Nucleic Acids Res Jan* **41** (Database issue):D348-352. doi: 10.1093/nar/gks1243. (http://www.ncbi .nlm.nih.gov/cdd).

8. Roy A, Kucukural A & Zhang Y (2010) I-TASSER: a unified platform for automated protein structure and function prediction. *Nat Protoc* **5**, 725–738 (<http://zhanglab.ccmb.med.umich.edu/I-TASSER>)

9. Zhang Y. (2007) Template-based modeling and free modeling by I-TASSER in CASP7. *Proteins* **69** (Suppl. 8), 108–117. (http://zhanglab.ccmb.med.umich.edu/I-TASSER).

10. Zhang Y. (2008) I-TASSER server for protein 3D structure prediction. *BMC Bioinformatics* **9**, 40. (http://zhanglab.ccmb.med.umich.edu/I-TASSER).

11. Blom N, Gammeltoft S & Brunak S. (1999) Sequence- and structure-based prediction of eukaryotic protein phosphorylation sites. *J Mol Biol* **294(5)**, 1351–1362. ([www.cbs.dtu.dk/services/NetPhos/](http://www.cbs.dtu.dk/services/NetPhos/)).

12. Ren J, Gao X, Jin C, *et al.* (2009) Systematic study of protein sumoylation: Development of a site-specific predictor of SUMOsp 2.0. *Proteomics* **9**, 3409–3412. (<http://sumosp.biocuckoo.org/online.php>)

13. Steentoft C, Vakhrushev SY, Joshi HJ, *et al.* (2013) Precision mapping of the human O-GalNAc glycoproteome through SimpleCell technology. *EMBO J* **32(10)**, 1478–1488.

14. Julenius K. (2007) NetCGlyc 1.0: Prediction of mammalian C-mannosylation sites. *Glycobiology* **17**, 868–876.

15. Johansen MB, Kiemer L & Brunak S. (2006) Analysis and prediction of mammalian protein glycation. *Glycobiology* **16**, 844–853.

16. Blom N, Sicheritz-Pontén T, Gupta R, *et al.* (2004) Prediction of post-translational glycosylation and phosphorylation of proteins from the amino acid sequence. *Proteomics* **4**, 1633–1649.

17. Bagos PG, Liakopoulos TD & Hamodrakas SJ (2006) Algorithms for incorporating prior topological information in HMMs: application to transmembrane proteins. *BMC Bioinformatics* **7**, 189. (<http://bioinformatics.biol.uoa.gr/HMM-TM/>).

18. Moller S, Croning MDR & Apweiler R (2001) Evaluation of methods for the prediction of membrane spanning regions. *Bioinformatics* **17(7)**, 646–653

(<http://www.cbs.dtu.dk/services/TMHMM/>).

19. Spyropoulos IC, Liakopoulos TD, Bagos PG, *et al.* (2004) TMRPres2D: high quality visual representation of transmembrane protein models. *Bioinformatics* **20**, 3258–3260 (http://bioinformatics.biol.uoa.gr/TMRPres2D/)

20. Nordahl Petersen T, Brunak s, von Heijne g, *et al.* (2011) SignalP 4.0: discriminating signal peptides from transmembrane regions.  *Nat. Methods* **8,** 785–786. (<http://www.cbs.dtu.dk/services/SignalP/>)

21. Yamashita A, Singh SK, Kawate T, *et al.* (2005) Crystal structure of a bacterial homologue of Na+/Cl--dependent neurotransmitter transporters. *Nature* **437(7056)**, 215–223.
